# Mieap suppresses murine intestinal tumor via its mitochondrial quality control

**DOI:** 10.1038/srep12472

**Published:** 2015-07-28

**Authors:** Masayuki Tsuneki, Yasuyuki Nakamura, Takao Kinjo, Ruri Nakanishi, Hirofumi Arakawa

**Affiliations:** 1 Division of Cancer Biology, National Cancer Center Research Institute, 5-1-1 Tsukiji, Chuo-ku, Tokyo 104-0045, Japan; 2 Division of Morphological Pathology, Department of Basic Laboratory Sciences, School of Health Sciences, Faculty of Medicine, University of the Ryukyus, 207 Uehara, Nishihara, Okinawa 903-0215, Japan

## Abstract

Mieap, a novel p53-inducible protein, plays a key role in maintaining healthy mitochondria in various pathophysiological states. Here, we show that Mieap deficiency in Apc^Min/+^ mice is strikingly associated with the malignant progression of murine intestinal tumors. To understand the role that Mieap plays in *in vivo* tumorigenesis, we generated Mieap heterozygous (Apc^Min/+^ Mieap^+/−^) and homozygous (Apc^Min/+^ Mieap^−/−^) Apc^Min/+^ mice. Interestingly, the Apc^Min/+^ mice with the Mieap^+/−^ and Mieap^−/−^ genetic background revealed remarkable shortening of the lifetime compared to Apc^Min/+^ mice because of severe anemia. A substantial increase in the number and size of intestinal polyps was associated with Mieap gene deficiency. Histopathologically, intestinal tumors in the Mieap-deficient Apc^Min/+^ mice clearly demonstrated advanced grades of adenomas and adenocarcinomas. We demonstrated that the significant increase in morphologically unhealthy mitochondria and trace accumulations of reactive oxygen species may be mechanisms underlying the increased malignant progression of the intestinal tumors of Mieap-deficient Apc^Min/+^ mice. These findings suggest that the Mieap-regulated mitochondrial quality control plays a critical role in preventing mouse intestinal tumorigenesis.

In the multistep tumorigenic processes of gastrointestinal tumors, the accumulation of unhealthy mitochondria followed by reactive oxygen species (ROS)-mediated mitochondrial dysfunction stimulates malignant conversion^1^. Recently, we identified a novel p53-inducible protein, Mieap (Mitochondria-eating protein), as a crucial regulator of a novel mitochondrial quality control (MQC) system^2^,^3^, which consists of two mechanisms, including a repair process and an elimination process. The molecular mechanism underlying this repair process is intriguing; MALM, the Mieap-induced accumulation of lysosomal proteins within mitochondria, leads to a striking decrease in mitochondrial reactive oxygen species (mtROS) generation and an increase in mitochondrial ATP synthesis activities through the elimination of oxidized mitochondrial proteins, and it differs completely from mitophagy (autophagosome-mediated autophagy)^2^,^3^,^4^. When MALM is inhibited, MIVs (Mieap-induced vacuoles) engulf and degrade the damaged, unhealthy mitochondria, behaving similarly to mitophagy^3^. Therefore, Mieap positively regulates mitochondrial quality by repairing or eliminating unhealthy mitochondria via MALM or MIV generation, respectively^2^,^3^,^4^. There is little doubt that the Mieap-mediated MQC function is critical for diverse physiological and pathophysiological conditions *in vitro*^2^,^3^,^4^,^5^.

Intestinal carcinoma is one of the leading causes of cancer-related deaths. The inactivation of adenomatous polyposis coli (APC) is an evoking event leading to the development of intestinal adenoma^6^,^7^; therefore, the Apc^Min/+^ mouse model is one of the best to investigate intestinal adenoma formation, which is directly implicated in human intestinal tumorigenic status^8^,^9^. To elucidate the involvement of the Mieap-regulated MQC function in tumorigenesis and malignant progression *in vivo*, in the present study, we utilized the Apc^Min/+^ murine intestinal tumor model^7^,^8^ and generated Mieap-deficient Apc^Min/+^ mice. We found that Mieap deficiency significantly accelerated the intestinal tumorigenic process, resulting in shorter lifespans and increased tumor multiplicity. Histopathologically, we confirmed the substantially increased number of intestinal high-grade adenomas and adenocarcinomas in the Mieap-deficient Apc^Min/+^ mice. Our results demonstrate that the loss of Mieap contributes remarkably to the malignant progression of intestinal tumors through the inactivation of the Mieap-mediated MQC function in Apc^Min/+^ mice.

## Results

### Mieap gene-deleted Apc^Min/+^ mice died earlier from severe anemia

**Figure 1:**
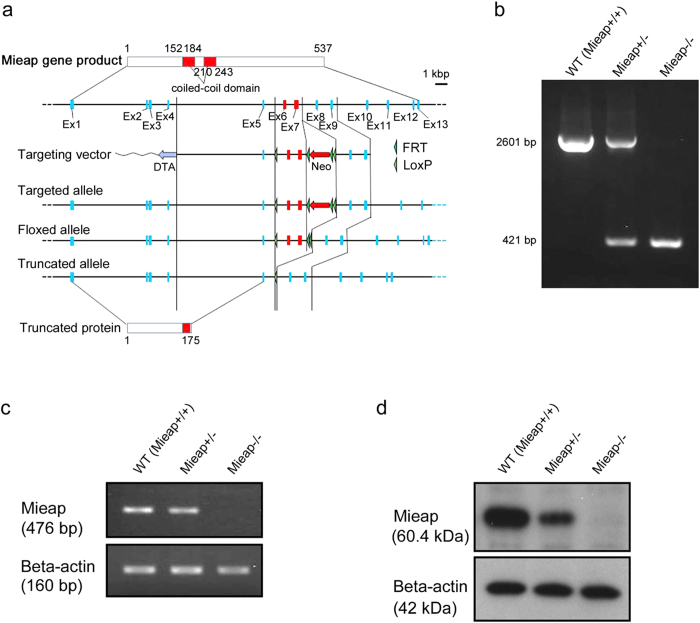
Generation of the Mieap-knockout mice. (**a**) The schematic diagram of the construction of Mieap gene mutant. Mieap-knockout mice were generated by using the Cre/loxP recombination system. Briefly, the floxed and trapped alleles were generated using a single construct bearing a gene-trap cassette doubly flanked by LoxP and FRT located between exons 5 and 8 of the mouse Mieap gene. (**b**) Genotypic analyses of the Mieap gene. The Genotypes were determined by PCR using genomic DNA derived from wild-type (WT), Mieap heterozygous mutant (Mieap^+/−^), and Mieap homozygous mutant (Mieap^−/−^) mice. (**c,d**) Mieap expression. Mieap mRNA (**c**) and protein (**d**) expressions were examined by RT-PCR and western blot in the testes from wild-type (WT), Mieap heterozygous mutant (Mieap^+/−^), and Mieap homozygous mutant (Mieap^−/−^) mice.

To investigate the *in vivo* role of Mieap, we generated the Mieap-knockout mice as shown in Fig. 1. WT, Mieap^+/−^, and Mieap^−/−^ mice were born in expected Mendelian ratios. We did not observe any developmental defects in the Mieap^+/−^ and Mieap^−/−^ mice. The Mieap^+/−^ and Mieap^−/−^ mice were normally born and were able to grow and live after birth as well. These results prompted us to speculate that Mieap deficiency may play a facilitatory role in tumorigenic/carcinogenic processes. Since the p53/Mieap-regulated mitochondrial quality control pathway is frequently inactivated in primary cancer tissues of colorectal cancer patients (manuscript in preparation), we examined the role of Mieap in the Apc^Min/+^ murine intestinal tumor model.

**Figure 2:**
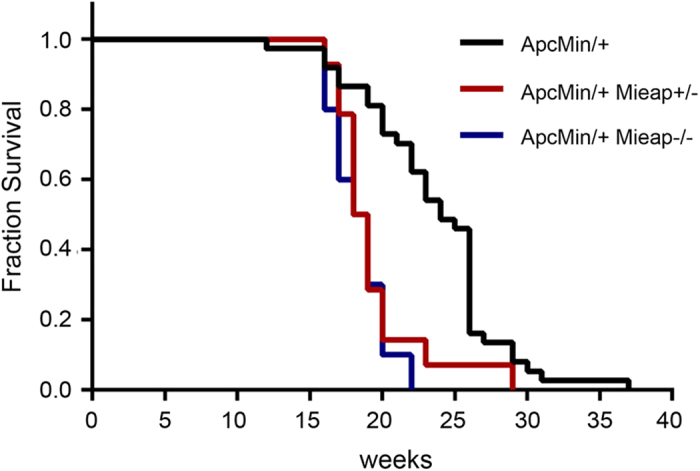
The Apc^Min/+^ Mieap^+/−^ and Apc^Min/+^ Mieap^−/−^ mice have shorter survival rates. The overall survival of the Apc^Min/+^ (n = 37), Apc^Min/+^ Mieap^+/−^ (n = 14), and Apc^Min/+^ Mieap^−/−^ (n = 10) mice from birth to death was plotted using the Kaplan-Meier method. The log-rank (Mantel-Cox) *P* values are as follows: Apc^Min/+^ vs. Apc^Min/+^ Mieap^+/−^, *P* = 0.0007 (statistically significant); Apc^Min/+^ vs. Apc^Min/+^ Mieap^−/−^, *P* < 0.0001 (statistically significant); Apc^Min/+^ Mieap^+/−^ vs. Apc^Min/+^ Mieap^−/−^, *P* = 0.4954.

**Figure 3:**
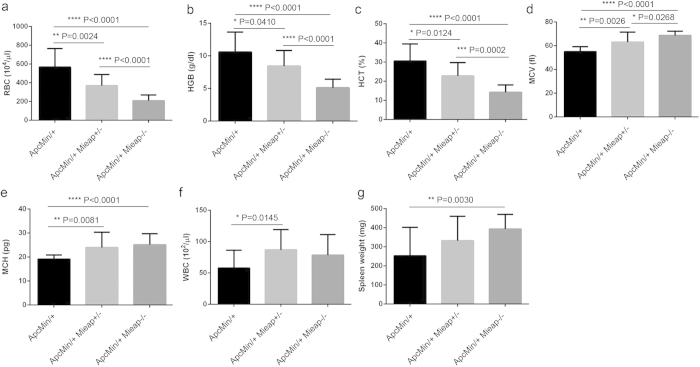
The Apc^Min/+^ Mieap^+/−^ and Apc^Min/+^ Mieap^−/−^ mice suffer much severer anemia compared to the Apc^Min/+^ mice. Hematological and blood chemical analyses for the red blood cell count (RBC) (**a**), hemoglobin (HGB) (**b**), hematocrit (HCT) (**c**), mean corpuscular volume (MCV) (**d**), mean corpuscular hemoglobin (MCH) (**e**), and white blood cell count (WBC) (**f**), as well as spleen weight (**g**) data, strongly indicated that the Apc^Min/+^ Mieap^+ /−,^ and Apc^Min/+^ Mieap^−/−^ mice tended to have much severer anemia, compared to the Apc^Min/+^ mice. The data are presented as the mean ± SD from seventeen-week-old Apc^Min/+^, Apc^Min/+^ Mieap^+/−^, and Apc^Min/+^ Mieap^−/−^ mice (n^ = ^15, each) (*P < 0.05; **P < 0.01; ***P < 0.001; ****P < 0.0001).

To investigate the role of Mieap in intestinal tumorigenesis *in vivo*, we generated the Apc^Min/+^ mice with the Mieap^+/−^ and Mieap^−/−^ genetic background, Apc^Min/+^ Mieap^+/−^ and Apc^Min/+^ Mieap^−/−^ mice. To evaluate the effects of Mieap gene deletion (heterozygous and homozygous deletions) on the overall survival of Apc^Min/+^ mice, we monitored a cohort of Apc^Min/+^ (n = 37), Apc^Min/+^ Mieap^+/−^ (n = 14), and Apc^Min/+^ Mieap^−/−^ (n = 10) mice until death. Interestingly, Kaplan-Meier survival analysis demonstrated that the Apc^Min/+^ Mieap^+/−^ and Apc^Min/+^ Mieap^−/−^ mice had significantly much shorter survival times than the Apc^Min/+^ mice [Fig. 2; Apc^Min/+^ vs. Apc^Min/+^ Mieap^+/−^, *P* = 0.0007; Apc^Min/+^ vs. Apc^Min/+^ Mieap^−/−^, *P* < 0.0001, log-rank (Mantel-Cox) test]. Hematological and blood chemical analyses clearly revealed that the Apc^Min/+^ Mieap^+/−^ and Apc^Min/+^ Mieap^−/−^ mice had much severer anemia compared to the Apc^Min/+^ mice (Fig. 3a–g) because of intestinal hemorrhage.

### An increased number of intestinal polyps in Mieap gene-deleted Apc^Min/+^ mice

**Figure 4:**
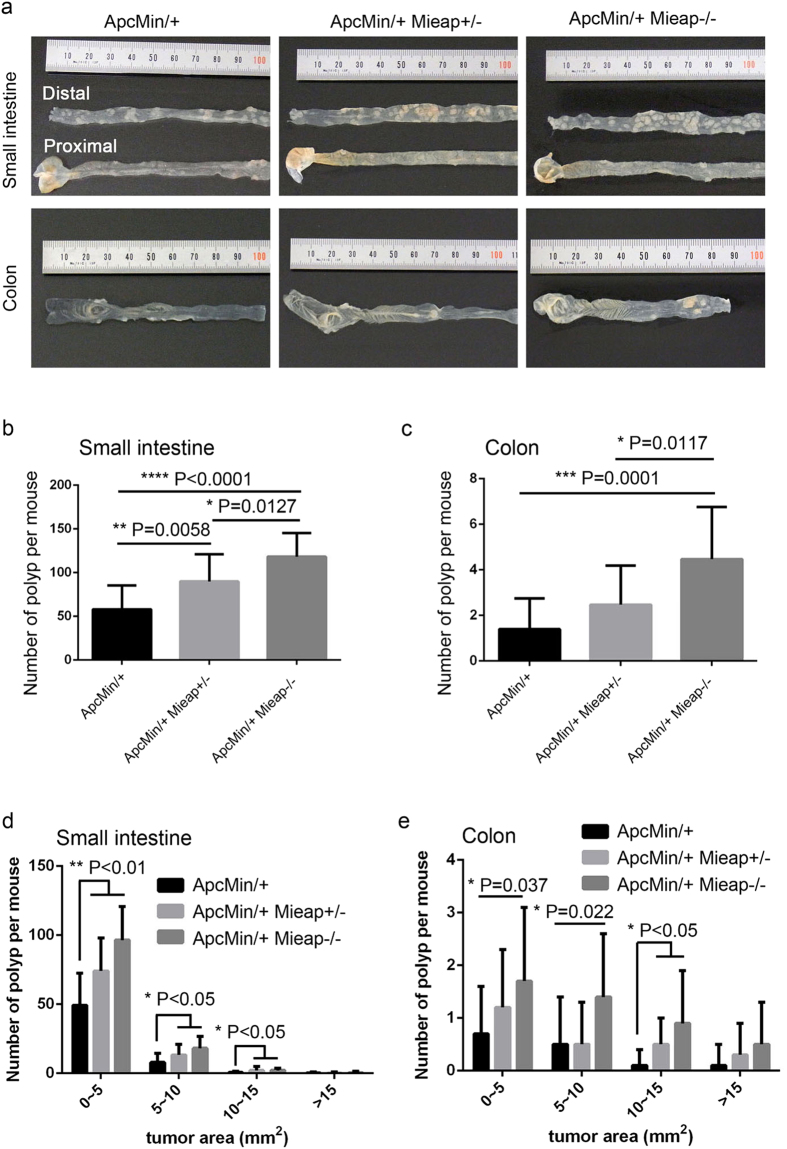
The number of intestinal polyps was increased in the Apc^Min/+^ Mieap^+/−^ and Apc^Min/+^ Mieap^−/−^ mice. (**a**) Representative examples of small intestines and colons from Apc^Min/+^, Apc^Min/+^ Mieap^+/−^, and Apc^Min/+^ Mieap^−/−^ mice. The substantially increased number of intestinal polyps in the small intestine (**b,d**) and colon (**c,e**) in the Apc^Min/+^ Mieap^+/−^, and Apc^Min**/**+^ Mieap^−/−^ mi**ce** compared to the Apc^Min/+^ mice. The data are presented as the mean ± SD from seventeen-week-old Apc^Min/+^, Apc^Min/+^ Mieap^+/−^, and Apc^Min/+^ Mieap^−/−^ mice (n = 15, each) (^*^P < 0.05; **P < 0.01; ^*^**P <^ ^0.001; ****P < 0.0001).

Since the Apc^Min/+^ mice die in anemia because of chronic intestinal tumor bleeding, we speculated that the Mieap-deficient Apc^Min/+^ mice (Apc^Min/+^ Mieap^+/–^ mice and Apc^Min/+^ Mieap^–/–^ mice) might burden much more tumors in the intestine. Therefore, we counted the number of intestinal polyps in the Apc^Min/+^ , Apc^Min/+^ Mieap^+/−^, and Apc^Min/+^ Mieap^−/−^ mice (n = 15, each) (Fig. 4). As shown in Fig. 4a–c, the Mieap gene-deleted Apc^Min/+^ mice (Apc^Min/+^ Mieap^+/−^ and Apc^Min/+^ Mieap^−/−^) had a greater number of intestinal polyps than the Apc^Min/+^ mice. Moreover, the intestinal polyps in the Mieap gene-deleted Apc^Min/+^ mice (Apc^Min/+^ Mieap^+/−^ and Apc^Min/+^ Mieap^−/−^) were significantly larger than those in the Apc^Min/+^ mice (Fig. 4d,e). These results suggest that Mieap plays a critical role in intestinal tumor suppression.

### Mieap gene deletion in Apc^Min/+^ mice promotes malignant tumor progression

To precisely evaluate the intestinal polyps in the Apc^Min/+^ , Apc^Min/+^ Mieap^+/−^, and Apc^Min/+^ Mieap^−/−^ mice (Fig. 4), all intestinal polyps were histopathologically diagnosed by two pathologists. In wild-type and Mieap^−/−^ mice, intestinal tumors were not observed, and there were few histological differences between these lines (Supplementary Fig. S1). Additionally, there were essentially no histological differences among the tumor-associated intestinal epithelia in the Apc^Min/+^, Apc^Min/+^ Mieap^+/−^, and Apc^Min/+^ Mieap^−/−^ mice. However, in the small intestines of the Apc^Min/+^ Mieap^−/−^ mice, the tumor-free epithelia were slightly atrophic (Supplementary Fig. S2).

**Figure 5:**
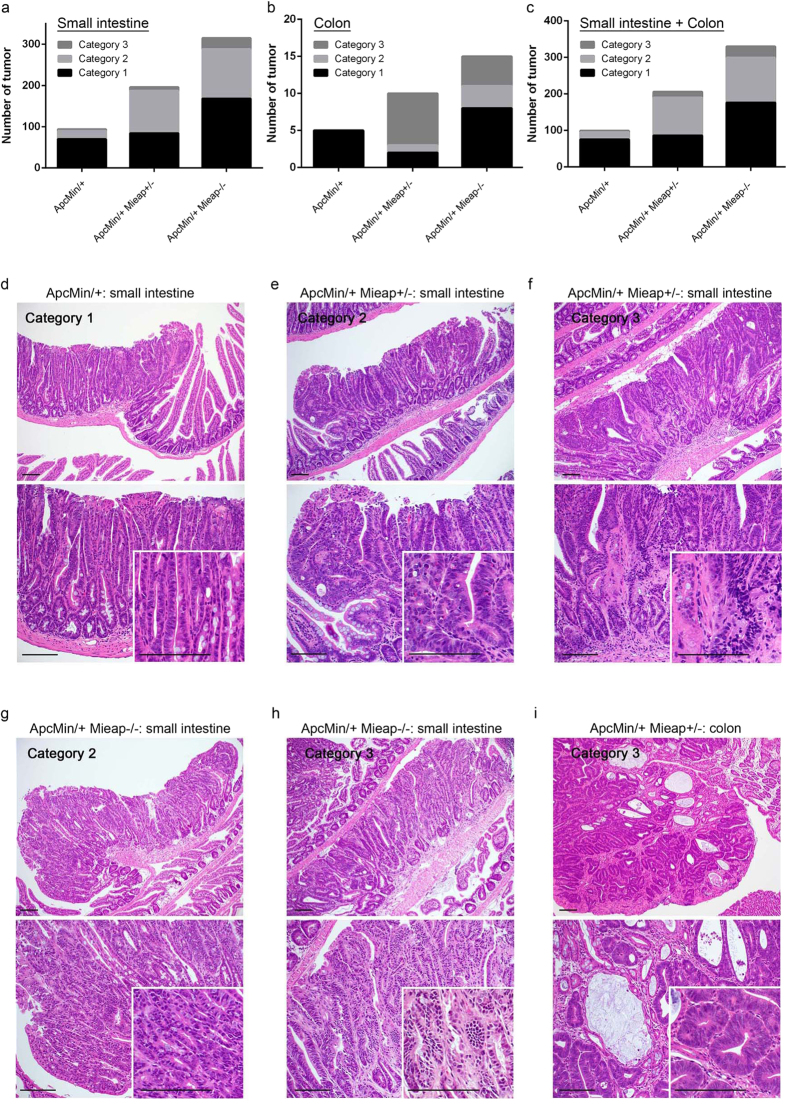
Substantial increase in the number of intestinal high-grade adenoma and adenocarcinoma in Apc^Min/+^ Mieap^+/−^ and Apc^Min/+^ Mieap^−/−^ mice. The numbers of histopathologically diagnosed small intestinal (**a**), colonic (**b**), and intestinal (small intestine plus colon) (**c**) adenomas and adenocarcinomas categorized into three classes (Category 1, low-grade adenoma; Category 2, high-grade adenoma; Category 3, adenocarcinoma) in the Apc^Min/+^, Apc^Min/+^ Mieap^+/−^, and Apc^Min/+^ Mieap^−/−^ mice (n = 8, each). Representative histopathology (HE) of Category 1 (low^-^grade adenoma) in the small intestines of an Apc^Min/+^ mouse (**d**), Category 2 (high-grade adenoma) (**e**) and Category 3 (adenocarcinoma) (**f**) in the small intestine of an Apc^Min/+^ Mieap^+/−^ mouse, Category 2 (high-grade adenoma) (**g**) and Category 3 (adenocarcinoma) (**h**) in the small intestine of an Apc^Min/+^ Mieap^−/−^ mouse, and Category 3 (adenocarcinoma) in the colons of the Apc^Min/+^ Mieap^+/−^ (**i**). (**d–i**) Top panel, low-power field; bottom panel with insets, high-power field. Scale bars, 100^ ^μm.

Neoplastic lesions among the intestinal polyps were categorized into three classes: Category 1, low-grade adenoma; Category 2, high-grade adenoma; and Category 3, adenocarcinoma (Fig. 5). The details of the definitions on each Category are described in the Methods section. We confirmed that the number of high-grade adenomas (Category 2) and adenocarcinomas (Category 3) in the small intestine (Fig. 5a), colon (Fig. 5b), and whole intestine (Fig. 5c) was substantially increased in accordance with the Mieap gene deletion in the Apc^Min/+^ mice (Fig. 5a–c). The majority of intestinal tumors in the Apc^Min/+^ mice were classified as Category 1, low-grade adenoma (Fig. 5a–c). Representative histopathology of a Category 1 tumor in the small intestine of Apc^Min/+^ mice is shown in Fig. 5d. There were distorted glandular architectures (Fig. 5d, upper panel) consisting of atypical cells with a modestly increased nuclear/cytoplasmic (N/C) ratio, but numerous goblet cells formed well-differentiated glandular structures (Fig. 5d, lower panel with inset). Focal penetration of the muscularis mucosae and crypt herniation were observed; however, the basement membrane remained intact (Fig. 5d).

In the Mieap gene-deleted Apc^Min/+^ mice, Category 1 intestinal tumors exhibited the same histology as that in Fig. 5d. In contrast to the Category 1 tumors, there were diversely shaped atypical/distorted glandular structures (Fig. 5e,g, upper panels) composed of dysplastic tumor cells with an increased N/C ratio (Fig. 5e,g, lower panels with insets) in the high-grade adenomas (Category 2) of the Apc^Min/+^ Mieap^+/−^ (Fig. 5e) and Apc^Min/+^ Mieap^−/−^ (Fig. 5g) mice. For Category 3, adenocarcinomas (Fig. 5f,h,i), there were apparent infiltrating irregularly shaped/sized tumor cell nests surrounded by fibrous and hyalinized stromata (Fig. 5f,h,i, lower panels with insets). Infiltrating tumor cells had large and dense nuclei and frequently exhibited less differentiated morphologies (Fig. 5f,h,i, lower panels with insets).

**Figure 6:**
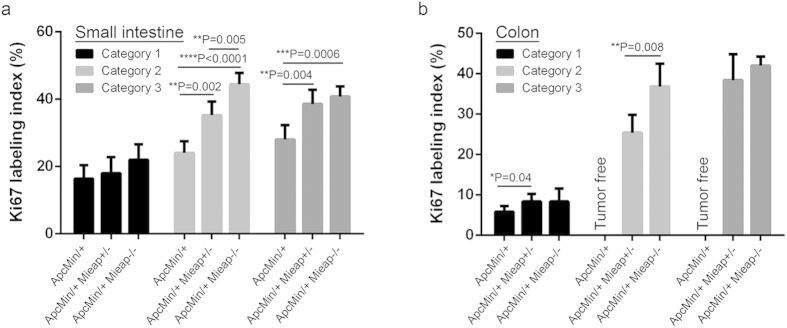
Higher tumor cell proliferative potentials in the intestinal adenomas and adenocarcinomas of the Apc^Min/+^ Mieap^+/−^ and Apc^Min/+^ Mieap^−/−^ mice. Small intestinal (**a**) and colonic (**b**) tumor cell proliferative potentials in the Apc^Min/+^, Apc^Min/+^ Mieap^+/−^, and Apc^Min/+^ Mieap^−/−^ mice (n = 8, each) were histopathologically evaluated by Ki67 labeling indices (%), which indicated statistically significant increases in the high-grade adenomas (Category 2) and adenocarcinomas (Category 3) in the Apc^Min/+^ Mieap^+/−^ and Apc^Min/+^ Mieap^−/−^ mice compared to the Apc^Min/+^ mice. The data represent the mean Ki67-labeling indices ± SD (*P < 0.05; **P < 0.01; ***P < 0.001; ****P < 0.0001).

Interestingly, there were many irregularly shaped glandular tumor cell nests with abnormal mucous retention in the adenocarcinomas stemming from the colon (Fig. 5i). Colonic adenocarcinoma (Category 3) in the Apc^Min/+^ Mieap^−/−^ mice showed the same histology as that in Fig. 5i. Moreover, to quantitatively confirm the increased tumor cell proliferative potentials in conjunction with the Mieap gene deletion in the Apc^Min/+^ mice, Ki67 labeling indices were determined in each category. It was obvious that the small intestinal high-grade adenoma (Category 2) and adenocarcinoma (Category 3) cell proliferative potentials of the Mieap gene-deleted Apc^Min/+^ mice were significantly higher than those of the Apc^Min/+^ mice (Fig. 6a). The colonic high-grade adenoma (Category 2) tumor cells in the Apc^Min/+^ Mieap^−/−^ mice had an enhanced proliferative capacity compared to that in the Apc^Min/+^ Mieap^+/−^ mice (Fig. 6b). Gross Ki67 labeling indices in each Category were shown in Supplementary Fig. S3.

### Unhealthy mitochondria and oxidative stress accumulate in the intestine and tumor of Mieap-deficient Apc^Min/+^ mice

**Figure 7:**
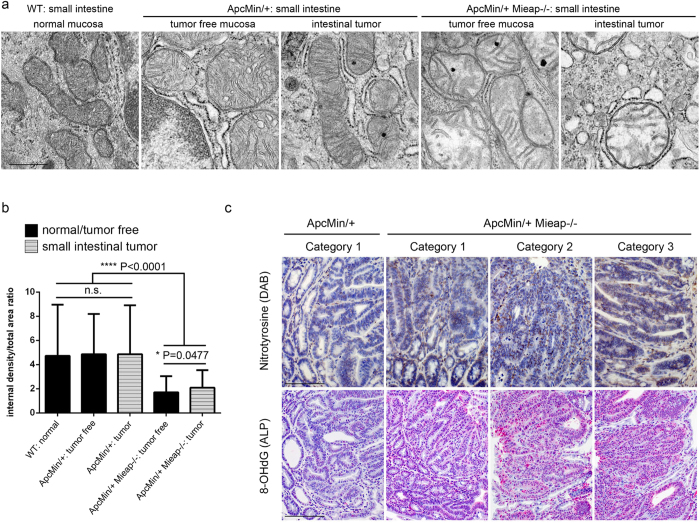
Unhealthy mitochondria and oxidative stress accumulate in the intestine and tumor of the Apc^Min/+^ Mieap^−/−^ mice. Electron microscopic analysis of the mitochondrial morphologies in wild-type (WT) normal mucosal epithelium (a, left panel), Apc^Min/+^ mice tumor-free mucosal epithelium (a, second from left panel) and small intestinal tumor cells (a, middle panel), Apc^Min/+^ Mieap^−/−^ mice tumor-free mucosal epithelium (a, second from right panel) and small intestinal tumor cells (a, right panel). Densitometric image analysis of the internal mitochondria (internal cristae density) was performed in the WT normal epithelium, Apc^Min/+^ tumor-free epithelium, Apc^Min/+^ tumor cells, Apc^Min/+^ Mieap^−/−^ tumor-free epithelium, and Apc^Min/+^ Mieap^−/−^ tumor cells in small intestine (**b**) (n = 100 mitochondria, each). In the tumor-free epithelial cells and small intestinal Apc^Min/+^ Mieap^−/−^ tumor cells, internal cristae density was morphologically (**a**) and statistically (**b**) decreased compared to that in the WT and Apc^Min/+^ mice mucosal epithelial cells and tumor cells. The data represent the mean internal density/total area ratio ± SD (*P < 0.05; ****P < 0.0001). (**c**) Immuno-peroxidase (DAB) staining for nitrotyrosine (upper row) and immuno-alkaline phosphatase (ALP) staining for 8-OHdG (bottom row), hematoxylin counterstain. Representative small intestinal tumor histopathology of Category 1 (low-grade adenoma) in Apc^Min/+^ (left column) and Apc^Min/+^ Mieap^−/−^ (second from left column) mice, Category 2 (high-grade adenoma, second from right column) and Category 3 (adenocarcinoma, right column) in the Apc^Min/+^ Mieap^−/−^ mice. Scale bars: 500 nm (a), 100 μm (c).

Since Mieap is a critical regulator of mitochondrial quality control^2^,^3^,^4^, and Mieap deficiency leads to an accumulation of unhealthy mitochondria and an increase in mtROS *in vitro*^2^,^3^, we speculated that the accumulation of unhealthy mitocnondria and upregulation of oxidative stress in the intestine and tumor may be involved in the mechanism for the promoted tumor malignant progression in the Mieap-deficient Apc^Min/+^ mice. Therefore, we examined the status of the mitochondria in the intestine and tumor of Apc^Min/+^ mice and the Apc^Min/+^ Mieap^−/−^ mice by performing electron microscopic analysis. As shown in Fig. 7a, compared with the small intestinal mucosal epithelial cells in the wild-type and Apc^Min/+^ mice, disruption of the cristae structure and integrity was remarkable in the Apc^Min/+^ Mieap^−/−^ mice. We performed the quantitative analysis on 100 mitochondria for mitochondrial density that reflects the healthy status of the cristae structure. Mitochondrial density in the tumor-free mucosal epithelial cells and tumor cells was extremely low due to crista defects in the Apc^Min/+^ Mieap^−/−^ mice (Fig. 7a,b). We confirmed that the same phenomenon occurred in the Mieap^−/−^ mice (Supplementary Fig. S4).

We further examined the status of oxidative stress using anti-nitrotyrosine antibody and anti-8-OHdG antibody to show the cytoplasmic and nuclear oxidative stresses, respectively. As shown in Fig. 7c, compared with the Apc^Min/+^ mice, there was a substantial increase in both nitrotyrosine and 8-OHdG immunopositivity in the small intestinal tumors of the Apc^Min/+^ Mieap^−/−^ mice (Fig. 7c) and the Apc^Min/+^ Mieap^+/−^ mice (Supplementary Fig. S5). These results suggest that the Mieap deficiency leads to the accumulation of unhealthy mitochondria in the intestine of the Apc^Min/+^ mice, and that unhealthy mitochondria are likely to increase oxidative stress, contributing to intestinal tumor progression.

## Discussion

According to our recent studies, Mieap is a critical regulator of mitochondrial quality control under pathophysiological conditions^2^,^3^,^4^. Therefore, Mieap deficiency leads to an accumulation of unhealthy mitochondria and an increase in mtROS^2^,^3^. It has been reported that mtROS generated by unhealthy mitochondria participate in genomic DNA insults^10^, stabilizing the oxygen-sensitive transcription factor (HIF1α)^11^,^12^, matrix metalloproteinase (MMP) induction^13^,^14^, and oxidative stress-related signaling pathways, including NF-κB^15^,^16^. Accordingly, we specifically focused on Mieap as a key regulator in suppressing mtROS-mediated tumor progression. Therefore, we first hypothesized that Mieap deficiency (global knockout) increased the susceptibility of the mice to spontaneous tumorigenesis/carcinogenesis in multiple organs; however, strikingly, there was no difference in spontaneous carcinogenesis or in the long-term survival rate compared to the wild-type mice, at least until two years after birth (manuscript in preparation). These results suggest that Mieap deficiency plays a facilitatory role in tumorigenic/carcinogenic processes.

One of the most useful and established animal models used to investigate intestinal tumor progression is the Apc^Min/+^ mouse model^8^,^17^. Apc^Min/+^ mice recapitulate human intestinal adenoma formation with germline mutations in APC (adenomatous polyposis coli) and provide a strong animal model for studying intestinal tumorigenesis^7^,^8^,^17^. To investigate the role of Mieap in intestinal tumor progression in Apc^Min/+^ mice, we generated heterozygous (Apc^Min/+^ Mieap^+/−^) and homozygous (Apc^Min/+^ Mieap^−/−^) Mieap gene-deleted Apc^Min/+^ mice. A substantially increased number of intestinal polyps (in the small intestines and colon) in Mieap-deficient mice compared to general Apc^Min/+^ mice indicated that Mieap is a key suppressor of intestinal tumor formation. Through histopathological analysis of each intestinal polyp, we confirmed that Mieap deficiency remarkably promoted tumorigenesis (excluding adenomatous/polypoid hyperplasia^18^,^19^) as well as malignant progression (adenocarcinoma). Moreover, Mieap deficiency triggered an enhanced proliferative capacity in intestinal adenoma and adenocarcinoma cells. As a possible explanation for abnormal intestinal tumor progression in Mieap-deficient Apc^Min/+^ mice, we demonstrated the accumulation of morphologically abnormal mitochondria revealing decreased cristae density^20^,^21^.

Unhealthy mitochondria produce higher levels of ROS^22^,^23^. This could be due to abnormal electron transfer by dysfunctional respiratory chain proteins; impaired ATP production by dysfunctional ATP synthase proteins; and/or the decreased supply of NADH resulting from dysfunctional TCA cycle proteins. The generated ROS also oxidize mitochondrial proteins, including the core proteins of energy production themselves, leading to a vicious cycle and the acceleration of mitochondrial dysfunction^22^,^23^. Consistent with this mechanism, we also demonstrated the accumulation of cytoplasmic and nuclear oxidative insults in the Mieap-deficient intestinal tumors, as indicated by nitrotyrosine^24^,^25^ and 8-OHdG^26^,^27^ immunohistochemistry. Therefore, these results support our hypothesis that the accumulation of unhealthy mitochondria promotes intestinal tumor formation and progression via increased mtROS generation.

The localization of APC in the epithelia of intestinal villi and colorectal crypts, which shows a pronounced gradient in its expression levels from nearly negative at the bottom of the crypts to strongly positive at the luminal side^28^,^29^,^30^, provides insight into the actual molecular function(s). APC negatively regulates cell proliferation in the intestine by suppressing the canonical Wnt signaling pathway^31^, which stimulates the TCF-dependent transcription of Wnt-target genes, such as c-MYC^32^, EphB/ephrinB^33^, and cyclin D1^34^, followed by β-catenin activation^35^. In addition to cell proliferation, APC inactivation has been reported to promote intestinal tumorigenesis through the downregulation of cell adhesion^36^,^37^. APC is associated with β-catenin, which links E-cadherin to α-catenin and the actin cytoskeleton, and positively regulates cell adhesion by controlling the distribution of β-catenin and E-cadherin between the cell membrane and cytoplasm/nucleus^38^,^39^. We also observed a strong cytoplasmic/nuclear localization of β-catenin in the intestinal tumor cells of the Mieap-deficient Apc^Min/+^ mice compared to the Mieap wild-type Apc^Min/+^ mice (manuscript in preparation). Therefore, it is possible that Mieap-mediated mitochondrial quality control is involved in the Wnt/β-catenin signaling pathway.

We have also paid particular attention to the Hippo pathway, which is a downstream signaling event of cell-to-cell interaction and critically regulates appropriate cell survival^40^,^41^. Therefore, uncontrolled cell proliferation due to the dysregulation of the Hippo pathway (Hippo-OFF) is directly responsible for tumorigenesis^42^,^43^,^44^. Recently, it has been reported that cross-talk between the Wnt/β-catenin and Hippo pathways played a crucial role in balanced cell growth in the early development process and tissue homeostasis^45^,^46^,^47^. These studies have led to the same conceptual framework positing that cell-to-cell interaction mediated by adhesion molecules critically controls appropriate cell proliferation and cell death. To explain the malignant progression mediated by Mieap deficiency in intestinal tumors, we speculated that there was crosstalk between Mieap-mediated mitochondrial quality control and the Hippo pathway because it has been reported that mitochondrial dysfunction stimulated ROS production and inactivated the Hippo pathway through c-Jun amino (N)-terminal kinase (JNK) signaling^48^.

Our present results strongly indicate that Mieap-mediated mitochondrial quality control plays a key role in suppressing intestinal tumorigenesis and malignant progression *in vivo*. Indeed, the Mieap-related pathway was inactivated in more than 70% of colorectal cancer patients (manuscript in preparation). Regarding the actual mechanism(s) for p53-mediated tumor suppression, there is still missing information^49^,^50^. Our findings emphasize that Mieap-mediated tumor suppression may be a possible candidate for one of the critical functions of p53 *in vivo*.

## Methods

### Animal ethics statement

All animal experimental protocols were approved by the National Cancer Center Animal Ethics Committee (approved protocol No. T11-031), and the animal experiments were conducted in accordance with the institutional guidelines for animal experiments, which meet the ethical standards required by the law and the guidelines concerning experimental animals in Japan.

### Mouse models

Wild-type (WT) C57BL6/J mice were obtained from CLEA Japan, Inc. (Tokyo, Japan). Apc^Min/+^ (C57BL/6J-Apc^Min^/J, Stock No. 002020) mice were obtained from The Jackson Laboratory (Bar Harbor, Maine, USA). The Mieap-knockout (Mieap^−/−^) mice were generated by using the *Cre/loxP* recombination system. Briefly, the floxed and trapped alleles were generated using a single construct bearing a gene-trap cassette doubly flanked by LoxP and FRT located between exons 5 and 8 of the mouse Mieap gene, which is located on chromosome 5. The Mieap homozygous (Mieap^−/−^) deficient mice were generated by mating between bleeding pairs of the Mieap heterozygous (Mieap^+/−^) mice. To determine the appropriate Mieap deficiencies, genomic DNA from the tails or fingers of the 3–4 week-old mice were genotyped by conventional genomic PCR using the Mieap knockout mice primers (forward, 5′-TCCCTTGAATCTTAACTTTGATGTC-3′; reverse, 5′-CTAAGACTGGCAGAAGACCAATAAG-3′). The Mieap expression was examined at mRNA and protein levels in the testes derived from the WT, Mieap^+/−^, and Mieap^−/−^ mice by RT-PCR (primers: Mieap forward, 5′-CGTGGAGACAATCAAGTGTC-3′; Mieap reverse, 5′-CAGCTATCTCTTCCTTCAGAT-3′; beta-2MG forward, 5′-TGGTGCTTGTCTCACTGACC-3′; beta-2MG reverse, 5′-CCGTTCTTCAGCATTTGGAT-3′) and western blot analysis (using rabbit polyclonal anti-mouse Mieap antibody and mouse monoclonal anti-beta actin antibody).

Mieap heterozygous (Apc^Min/+^ Mieap^+/−^) and homozygous (Apc^Min/+^ Mieap^−/−^) deficient Apc^Min/+^ mice were generated by mating between Apc^Min/+^ mice and the Mieap-deficient mice (Mieap^+/−^ or Mieap^−/−^ mice). To determine the appropriate Mieap deficiencies on Apc^Min/+^ mice, genomic DNA from the tails or fingers of the 3–4 week-old mice were genotyped by conventional genomic PCR using Apc^Min/+^ mice primers (The Jackson Laboratory protocol; wild-type forward, 5′-GCCATCCCTTCACGTTAG-3′; Min forward, 5′-TTCTGAGAAAGACAGAAGTTA-3′; common reverse, 5′-TTCCACTTTGGCATAAGGC-3′) and the Mieap knockout mice primers.

### Survival studies

The overall survival of Apc^Min/+^ (n = 37), Apc^Min/+^ Mieap^+ /−^ (n = 14), and Apc^Min/+^ Mieap^−/−^ (n = 10) mice was calculated from birth to the ethical end point or death. Survival distribution was estimated using the Kaplan-Meier overall survival method.

### Surgical procedures

Seventeen-week-old Apc^Min/+^, Apc^Min/+^ Mieap^+/−^, and Apc^Min/+^ Mieap^−/−^ mice (n = 15, each) were anesthetized using diethyl ether. Peripheral blood samples collected from the eye socket using K2EDTA capillary tubes (VITREX Medical, Herlev, Denmark) were hematologically analyzed for red blood cells (RBC), hemoglobin (HGB), hematocrit (HCT), mean cell volume (MCV), mean cell hemoglobin (MCH), and white blood cells (WBC) (CLEA Japan). The number and size of the intestinal polyps were calculated under a SMZ-10 stereoscopic microscope (Nikon, Tokyo, Japan).

### Transmission electron microscopy

The small intestinal specimens were cut 1 mm^3^ from the WT, Apc^Min/+^, and Apc^Min/+^ Mieap^−/−^ mice and were fixed in cold 2% paraformaldehyde/glutaraldehyde in 0.1 M PBS (pH 7.2) at 4 °C for 16 hours. After being rinsed with 0.1 M PBS at 4 °C for 90 min, the specimens were fixed in cold 1% osmium-tetroxide in 0.1 M PBS and then dehydrated with ethanol at 4 °C (from 50% to 100%: 10 min, each). Finally, the specimens were embedded in EPON 812 (TAAB Laboratories Equipment Ltd., Berks, England), and ultrathin sections were cut at 70 nm using a Leica UltraCut UCT microtome (Leica Microsystems, Wetzlar, Germany) and stained with uranium-lead. Digital electron microscopy images were captured using the H-7500 transmission electron microscope (HITACHI, Tokyo, Japan). The digital images were analyzed using ImageJ 1.49o software (National Institutes of Health, USA) on a Windows 7 computer.

### Specimen handling

Intestinal (small intestinal and colonic) specimens were collected from 17-week-old WT, Mieap^−/−^, Apc^Min/+^, Apc^Min/+^ Mieap^+/−^, and Apc^Min/+^ Mieap^−/−^ mice (n = 8 each). The Swiss-roll intestinal surgical samples were routinely fixed in 10% formalin and embedded in paraffin. Serial 5 μm sections were cut from paraffin blocks and used for the histopathological studies.

### Hematoxylin and eosin (HE) staining and histopathological evaluation

HE staining was performed using Dako Eosin (Code CS701, Dako, Glostrup, Denmark) and Dako REAL^TM^ Hematoxylin (Code S2020, Dako)^51^,^52^. Histopathologically, all intestinal specimens were evaluated by two pathologists (MT and TK), and the intestinal epithelial tumors (polyps) were categorized into the following three classes: Category 1, low-grade adenoma; Category 2, high-grade adenoma; and Category 3, adenocarcinoma. The defining histopathological features for each category are as follows. Category 1: mildly distorted glandular structures, branching villi and tubular crypt proliferation, mild nuclear and cellular atypism, and intact basement membrane; Category 2: moderately or severely distorted glandular structures with branching villi, severe nuclear and cellular atypism, increased mitotic figures, increased atypical mucous retention, and intact basement membrane; Category 3: evident infiltration of tumor cell nests with tumor stromal induction, increased mitotic figures, penetration of the basement membrane, and invasion of the lamina propria or muscularis mucosae.

### Antibodies

Rabbits were immunized with the recombinant amino (N)-terminal domain of mouse Mieap protein. Rabbit polyclonal anti-mouse Mieap antibody was subsequently purified on antigen affinity columns. Rabbit polyclonal antibodies against mouse Ki67 (catalog no. ab15580) were purchased from Abcam (Cambridge, MA, USA). A mouse monoclonal antibody against mouse 8-hydroxy-2′-deoxyguanosine (8-OHdG) (clone: N45.1, catalog no. MOG-100P)^53^ was purchased from the Japan Institute for the Control of Aging (JaICA), NIKKEN SEIL Co., Ltd (Shizuoka, Japan). A mouse monoclonal antibody against mouse nitrotyrosine (clone: 39B6, catalog no. NB110-96877) was purchased from Novus Biological USA (Littleton, CO, USA). A mouse monoclonal antibody against beta-actin (catalog no. A5316) was purchased from Sigma-Aldrich (St Louis, MO, USA).

### Immunohistochemistry

Immunohistochemistry was performed using the EnVision^TM^ + Dual Link System-HRP and EnVision^TM^ G|2 System/AP Rabbit/Mouse (Permanent Red) system (Dako). For 8-OHdG immunohistochemistry, we selected alkaline phosphatase (ALP) staining to avoid non-specific antigen-antibody reaction by hydrogen peroxide. For antigen retrieval, sections were autoclaved in citric acid buffer (pH 6.0) at 121 °C for 10 min^52^,^54^. The sections were treated with 0.3% hydrogen peroxide in methanol for 30 min at room temperature to block endogenous peroxidase activity (for Ki67 and nitrotyrosine) and incubated with 5% bovine serum albumin (BSA) in 50 mM Tris-buffered saline (pH 7.4) containing 0.05% Triton X-100 (T-TBS) for 1 hour at room temperature to block non-specific protein binding sites^54^. The sections were then incubated at 4 °C with the primary antibodies diluted at 1:20 (anti-8-OHdG), 1:50 (anti-Nitrotyrosine) and 1:1000 (anti-Ki67) in T-TBS. After overnight incubation, the sections were incubated with EnVision^TM^ + Dual Link System-HRP reagents for 1 hour or EnVision^TM^ G|2 System/AP Rabbit/Mouse (Permanent Red) system reagents according to the manufacturer’s instruction at room temperature and treated with 0.02% 3,3′-diaminobenzidine, tetrahydrochloride (CAS no. 7411-49-6, catalog no. D006, DOJINDO LABORATORIES, Kumamoto, Japan) in 0.05 M Tris-HCl buffer (pH 7.6) containing 0.005% hydrogen peroxide or permanent red reagent according to the manufacturer’s instructions to visualize the reaction products^54^. Finally, sections were counterstained with Dako REAL^TM^ Hematoxylin (Dako)^51^. Digital HE and immunohistochemical images were captured on an Olympus BX43 microscope equipped with a DP27 digital camera and a D21-SAL stand-alone unit (Olympus Corporation, Tokyo, Japan) and compiled with Photoshop CS2 software (Adobe Systems Software Ireland Ltd., San Jose, CA, USA) on a Windows 7 computer.

### Ki67 labeling index

The percentage of tumor cells with Ki67-positive nuclei among 100 cells was calculated in five fields of each intestinal tumor by pathologists, and the mean ± standard deviation (SD) values were determined as Ki67-labeling indices^55^.

### Statistical analysis and preparing graphs

All graphs and statistical analysis of all the experiments in this study were performed using GraphPad Prism version 6.03 for Windows (GraphPad Software Inc., La Jolla, CA, USA)^41^,^43^. Kaplan-Meier analysis and log-rank (Mantel-Cox) tests were used to compare the survival curves^56^. Other statistical analyses were performed using an unpaired Student’s *t*-test. P-values of less than 0.05 were considered to be significant^41^,^43^. Significant *P*-values are provided in the figure panels and result sections.

## Additional Information

**How to cite this article**: Tsuneki, M. *et al.* Mieap suppresses murine intestinal tumor via its mitochondrial quality control. *Sci. Rep.* **5**, 12472; doi: 10.1038/srep12472 (2015).

## Supplementary Material

Supplementary Information
