# Supplementary material for: Mieap suppresses murine intestinal tumor via its mitochondrial quality control
Source: Sci Rep. 2015 Jul 28;5:12472. doi: 10.1038/srep12472 (PMC4516962; doi:10.1038/srep12472)
Supplement: Supplementary Information [file srep12472-s1.pdf]

Supplementary Information

Mieap suppresses murine intestinal tumor via its mitochondrial quality control

Masayuki Tsuneki[1], Yasuyuki Nakamura[1], Takao Kinjo[2], Ruri Nakanishi[1], and Hirofumi Arakawa[1]

[1]Division of Cancer Biology, National Cancer Center Research Institute, 5-1-1 Tsukiji, Chuo-ku, Tokyo 104-0045, Japan
[2]Division of Morphological Pathology, Department of Basic Laboratory Sciences, School of Health Sciences, Faculty of Medicine, University of the Ryukyus, 207 Uehara, Nishihara, Okinawa, 903-0215, Japan

Figure S1. Histopathology of the intestinal mucosal epithelia in the wild-type and Mieap$^{-/-}$ mice.

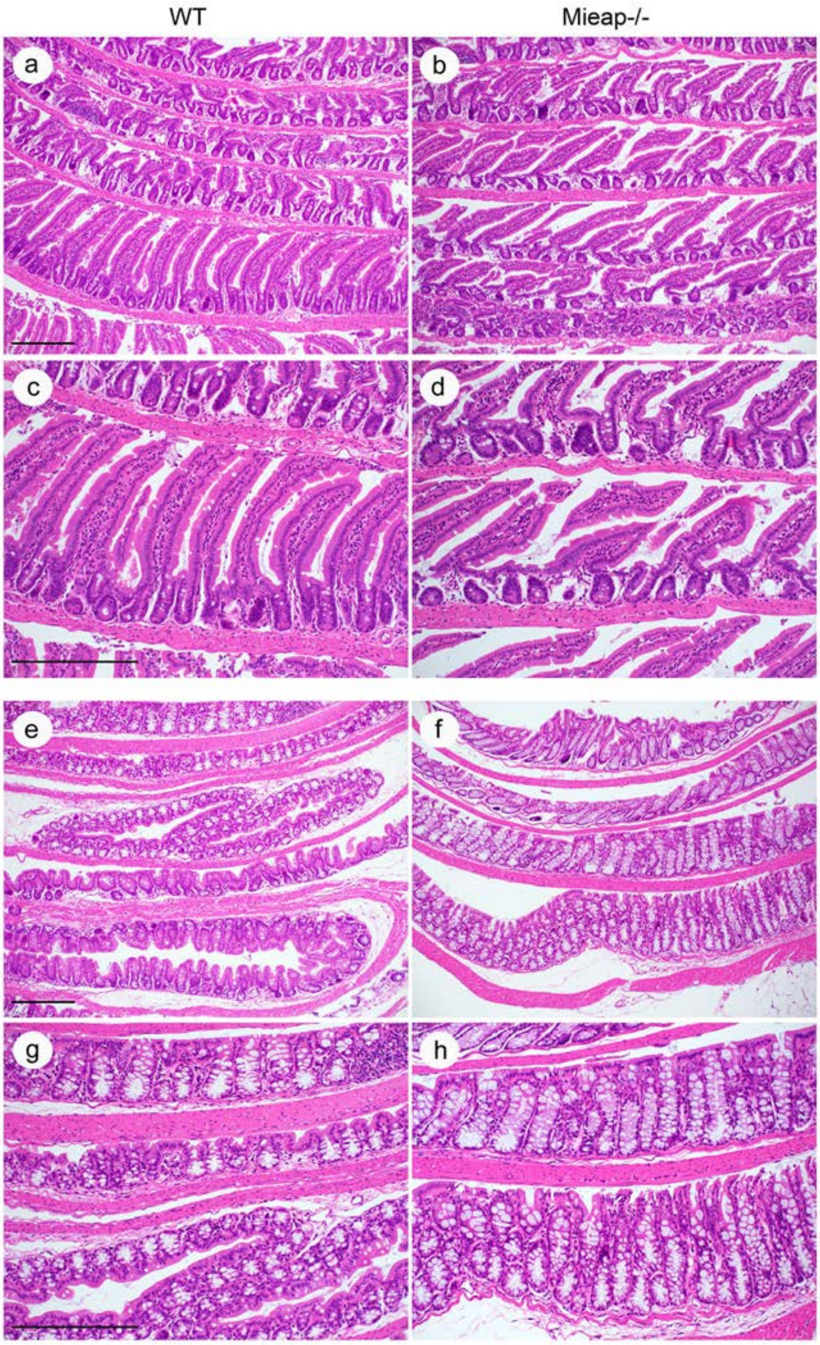

Microscopic images of small intestinal epithelia (a-d) and colonic epithelia (e-h) in the wild-type (WT) (a, c, e, g) and Mieap$^{-/-}$ (b, d, f, h) mice. Scale bars, 200 μm. No polyps/tumors could be found, and the histology was nearly normal in the small intestines and colons of the WT and Mieap$^{-/-}$ mice.

Figure S2. Histopathology of tumor-free intestinal mucosal epithelia in the $Apc^{Min/+}$, $Apc^{Min/+}Mieap^{+/-}$, and $Apc^{Min/+}Mieap^{-/-}$ mice.

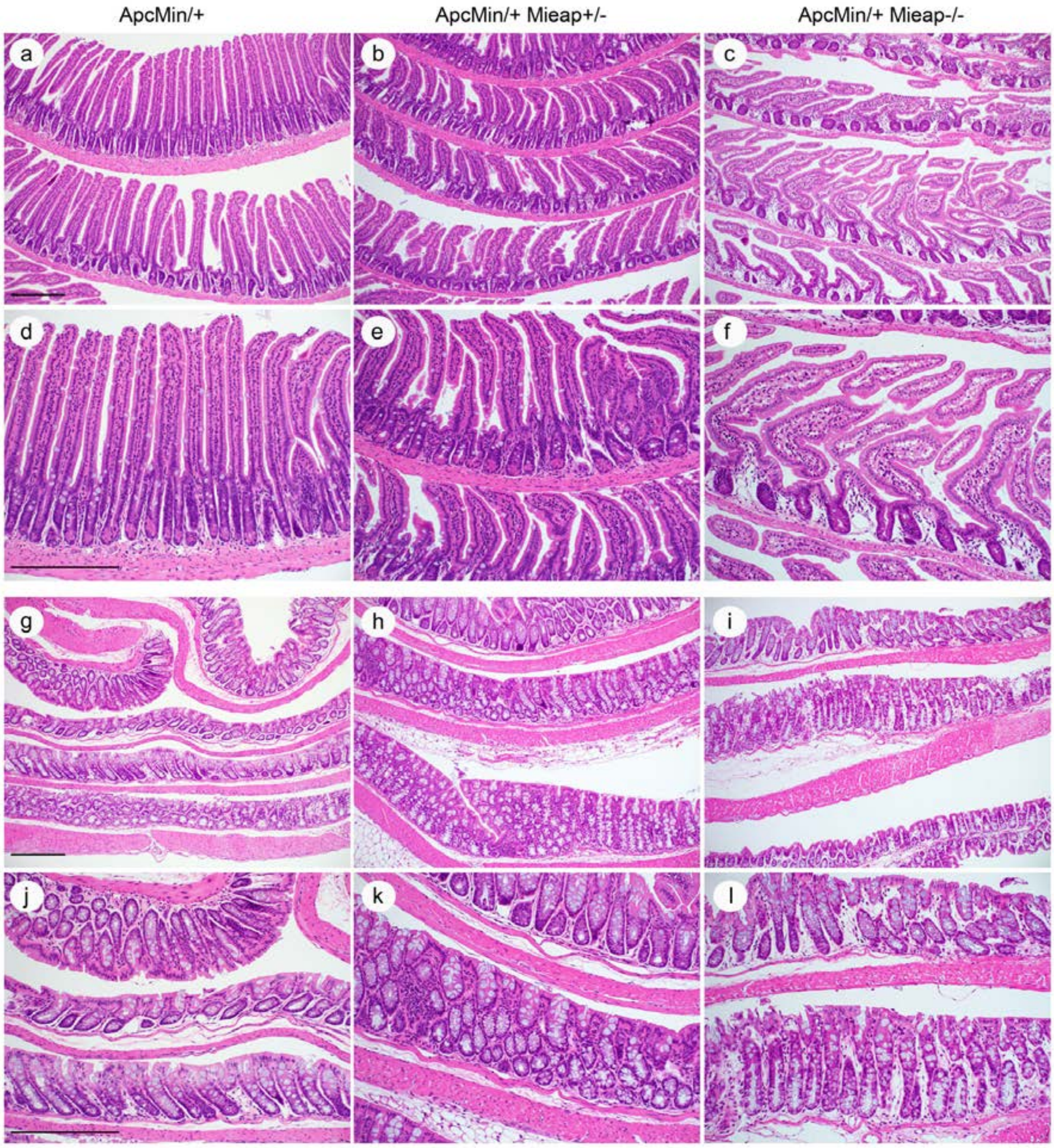

Microscopic images of small intestinal epithelia (a-f) and colonic epithelia (g-l) in $Apc^{Min/+}$ (a, d, g, j), $Apc^{Min/+}Mieap^{+/-}$ (b, e, h, k), and $Apc^{Min/+}Mieap^{-/-}$ (c, f, i, l). Scale bars, 200 μm. There were nearly no histopathological differences among the intestinal epithelia of the $Apc^{Min/+}$, $Apc^{Min/+}Mieap^{+/-}$, and $Apc^{Min/+}Mieap^{-/-}$ mice in terms of tumors. However, in the small intestines of the $Apc^{Min/+}Mieap^{-/-}$ mice, the tumor-free epithelia were slightly atrophic.

Figure S3. Robust increased Ki67 positive proliferating tumor cells in the high grade adenoma (Category 2) and adenocarcinoma (Category 3) as compared to the low grade adenoma (Category 1).

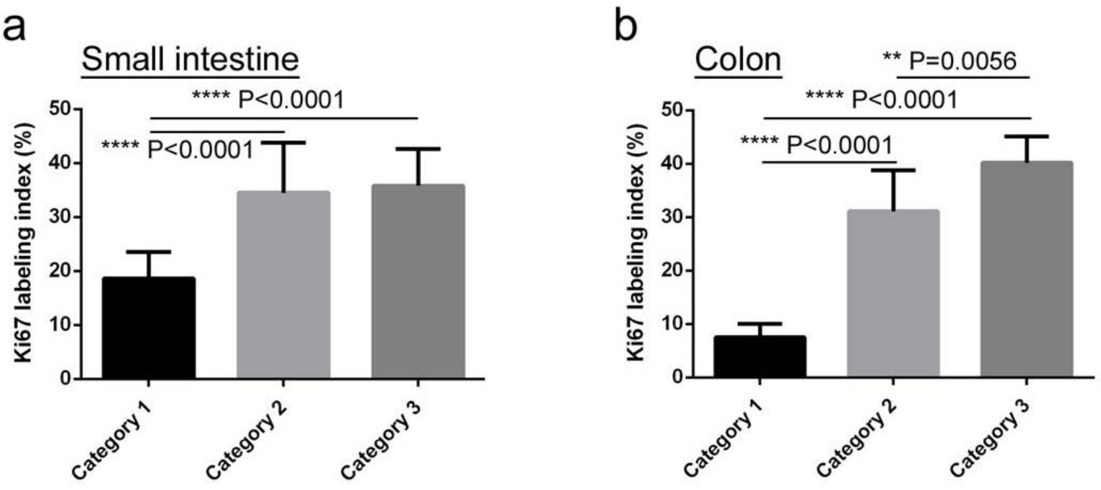

Small intestinal (a) and colonic (b) tumor cell proliferative potentials of the Category 1, 2, and 3 neoplasms were evaluated by Ki67 labeling indices (%).The data represent the mean Ki67-labeling indices ± SD (**, $P < 0.01$; ****, P < 0.0001).

Figure S4. Decreased internal mitochondrial density in the small intestinal mucosal epithelium of Mieap$^{-/-}$ mice.

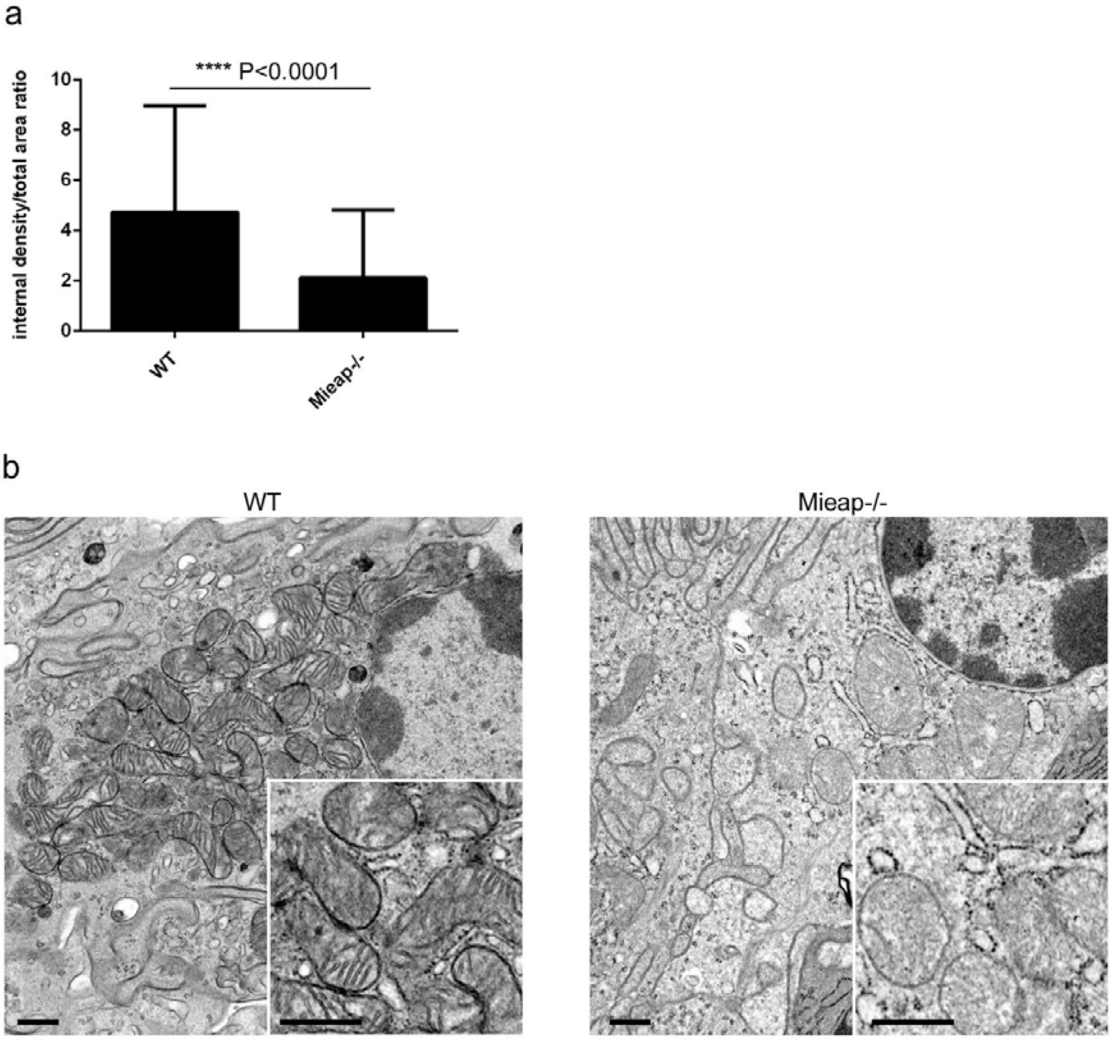

(a) Densitometric image analysis of the internal mitochondrial density (internal cristae density) was performed in the WT and Mieap$^{-/-}$ small intestinal mucosal epithelium (n = 100 mitochondria, each). The data represent the mean internal density/total area ratio ± SD (****, $P < 0.0001$). (b) Representative electron micrographs of mitochondrial architectures in WT (left panel with high power inset) and Mieap$^{-/-}$ (right panel with high power inset) mucosal epithelial cells. Scale bars, 500 nm.

Figure S5. 8-OHdG and nitrotyrosine immunolocalization in small intestinal tumors of the $Apc^{Min/+}Mieap^{+/-}$ mice.

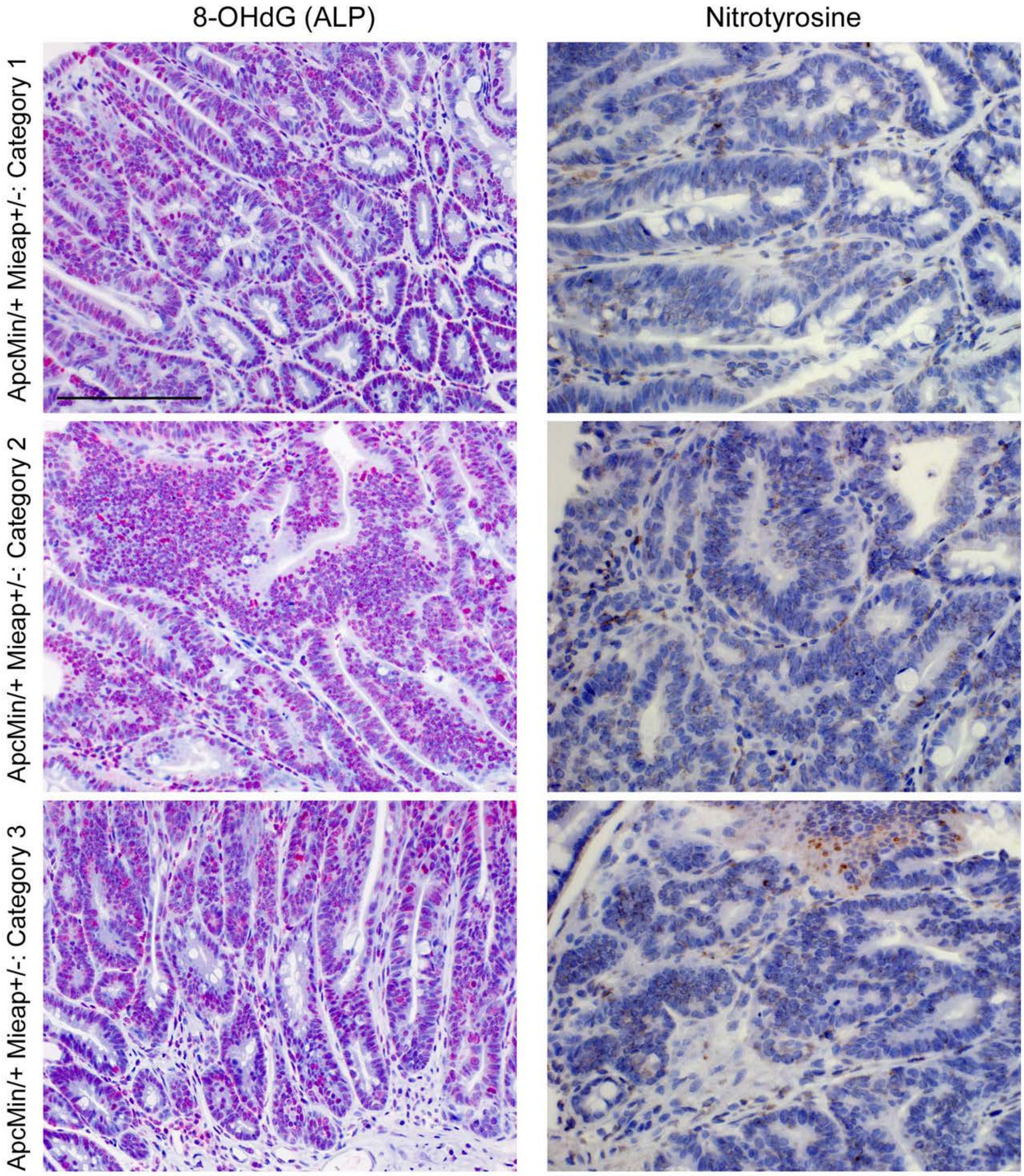

Immuno-alkaline phosphatase (ALP) staining for 8-OHdG (left column) and immuno-peroxidase (DAB) staining for nitrotyrosine (right column), hematoxylin counterstaining. Representative histology of Category 1 (top row), Category 2 (second from top row), and Category 3 (bottom row) in $Apc^{Min/+}Mieap^{+/-}$ mice. Scale bar, 100 μm.
